# Editorial to “Characteristics of radiofrequency lesions in patients with symptomatic periesophageal vagal nerve injury after pulmonary vein isolation”

**DOI:** 10.1002/joa3.13057

**Published:** 2024-05-12

**Authors:** Koichiro Ejima

**Affiliations:** ^1^ Department of Cardiology Minamino Cardiovascular Hospital Tokyo Japan

**Keywords:** atrial fibrillation, catheter ablation, complication, periesophageal vagal nerve injury, pulmonary vein isolation

Because the left atrium (LA) and esophagus are adjacent to each other, collateral damage to the periesophageal vagal nerve after ablation of atrial fibrillation (AF) is not uncommon.^1^ The left vagal nerve branches form a plexus anterior to the esophagus and control the esophageal and gastric motility, maintain a lower esophageal sphincter tone, and induce pyloric relaxation. Damage to the periesophageal nerve fibers may result in both reflux (facilitating progression of esophageal lesions) and gastric motility disorders/food retention. The exact pathophysiologic mechanisms of vagal nerve injury are not well understood. Besides the direct thermic effects on neural action potentials, edema and hematomas (disruption of vessels supplying the esophageal wall and giving rise to necrosis) may cause local pressure on vagal nerve branches.

In this issue of the *Journal of Arrhythmia*, Yoshimura et al. provided an important assessment of the relationship between the incidence of symptomatic periesophageal vagal nerve injury (PNI) during radiofrequency (RF) catheter ablation of AF and the RF lesion characteristics and distance between the RF lesions and esophagus.^1^ Of 1391 patients who underwent a first‐time ablation index‐guided pulmonary vein isolation (PVI) using a CARTO system for AF, 10 (0.72%) were diagnosed with symptomatic PNI. In that study, the ablation procedure was performed after integrating the LA electroanatomical maps with the computed tomography (CT) images obtained preprocedure. On the LA posterior wall near the esophagus, they restricted RF applications to a power setting of <30 W and RF duration of <30 s, regardless of the ablation index. Further, the RF delivery was stopped when the esophageal temperature (ET) reached >41°C. They found that the contact force (CF) at the lesion‐esophageal distance (LED), defined as the shortest perpendicular distance from the RF‐lesion tag on the circumferential ablation line to the anterior aspect of the esophagus, of 0–5 mm was an independent predictor of symptomatic PNI using a multivariate logistic analysis. It is known that the proximity of LA posterior wall to the esophagus is associated with esophageal injury. The clinical significance of this study was that it revealed that not only the proximity of the esophagus to the LA posterior wall but also the characteristics of the RF lesions, not the ablation index or RF power but the CF, were associated with symptomatic PNI. The fact that the LEDs in this study were not necessarily accurate and may have underestimated the PNI because the assessment of the PNI was dependent on the presence or absence of symptoms, was a limitation that should be noted when interpreting this study's results. Grosse Meininghaus et al.^2^ reported that PVI‐induced PNI and gastric motility disorders detected by electrogastrography are quite common and are observed in one‐third of patients postthermal energy PVI. Further, from their study result that PNI was part of the (peri)‐esophageal damage and only partially overlapped with the endoscopic findings, they stated that PNI‐associated acidic reflux may be involved in the complex pathophysiology of esophageal lesions progressing to fistulae.

Pulsed field ablation (PFA) creates myocardial tissue‐specific lesions by forming micropores in cell membranes, without risk of injury to collateral structures, including esophageal tissue. A recent study that assessed esophageal and periesophageal injury (mucosal lesions, food retention, periesophageal edema, and vagal nerve injury) using endoscopy, endoscopic ultrasound, electrogastrography,^2^ and cardiac magnetic resonance^3^ showed no signs of esophageal or periesophageal injury post‐PFA‐PVI. If PFA becomes widely used clinically, esophageal‐related complications associated with AF ablation may become a thing of the past. However, in the current situation where AF ablation using thermal energy must be performed, we must take utmost care and use our ingenuity to prevent esophageal‐related complications.

It is important to visualize the esophagus intraoperatively by image integration of the preoperative CT image and intraoperative LA map, as done in this study, but it should be noted that the esophagus's position can vary due to immediate prior positional changes.^4^

Luminal ET monitoring, also performed in this study, is an inadequate method to guide ablation therapy to avoid esophageal injury. It should be noted that the ET rises with a delay even after stopping the RF application, and the ET can be underestimated if the ablation catheter and temperature probe are not in close proximity. Moreover, in some cases, the esophageal probe itself may serve as an RF “antenna” and promote esophageal thermal injury.

Conscious sedation is reported to cause significantly less esophageal tissue damage post‐AF ablation than general anesthesia. The presumed mechanism is that general anesthesia reduces esophageal motility and eliminates patient swallowing, preventing physiological cooling.

Some reports have shown the usefulness of esophageal active cooling devices and mechanically displacing the esophagus to prevent esophageal injury, but both have only been utilized under general anesthesia.

Further ablation on the LA posterior wall added to the PVI is reported to be associated with esophageal thermal injury during RF catheter ablation. The usefulness of additional empirical ablation to the PVI has not been established for persistent or paroxysmal AF, and it should not be performed uniformly from the standpoint of preventing complications.

High‐power short‐duration (HPSD) RF ablation creates shallow, wide, and durable lesions during the resistive heating phase and reduces collateral tissue damage by shortening the conductive heating phase, which can generate a deeper lesion. A randomized controlled study showed that esophageal‐related complications are rare with HPSD RF AF ablation with or without ET monitoring. In my experience with HPSD RF AF ablation using CF‐sensing catheter guided by electroanatomical mapping (CARTO) combined with image integration without ET monitoring in more than 1000 patients, only one case had a symptomatic gastric motility disorder post‐ablation, and the patient recovered 1 week after conservative treatment. To achieve a safe and effective HPSD RF ablation, RF applications on the LA posterior wall adjacent to the esophagus should be limited to a CF <10 g during RF applications with minimal respiratory variability in the CF and with an RF application duration of <5 s and interlesion distance of <5 mm.^5^

## FUNDING INFORMATION

None.

## CONFLICT OF INTEREST STATEMENT

The authors declare no conflict of interest for this article.

## References

[1] Yoshimura S , Take Y , Kaseno K , Goto K , Matsuo Y , Aoki H , et al. Characteristics of radiofrequency lesions in patients with symptomatic periesophageal vagal nerve injury after pulmonary vein isolation. Journal of Arrhythmia. 2024. doi: 10.1002/joa3.13048. (in press). PMC11199810 38939771

[2] Grosse Meininghaus D , Freund R , Kleemann T , Geller JC , Matthes H . Pulmonary vein isolation‐induced vagal nerve injury and gastric motility disorders detected by electrogastrography: the side effects of pulmonary vein isolation in atrial fibrillation (SEPIA) study. J Cardiovasc Electrophysiol. 2023;34:583–592. 36640436 10.1111/jce.15820

[3] Cochet H , Nakatani Y , Sridi‐Cheniti S , Cheniti G , Ramirez FD , Nakashima T , et al. Pulsed field ablation selectively spares the oesophagus during pulmonary vein isolation for atrial fibrillation. Europace. 2021;23:1391–1399. 33961027 10.1093/europace/euab090 PMC8427383

[4] Ejima K , Shoda M , Futagawa K , Kimura R , Manaka T , Hagiwara N , et al. Transverse shifting of the esophagus according to the patient's position helped achieve a safe and successful pulmonary vein isolation procedure. Heart Vessels. 2009;24:317–319. 19626407 10.1007/s00380-008-1122-1

[5] Yazaki K , Ejima K , Kataoka S , Kanai M , Higuchi S , Yagishita D , et al. Regional differences in the predictors of acute electrical reconnection following high‐power pulmonary vein isolation for paroxysmal atrial fibrillation. J Arrhythm. 2021;37:1260–1269. 34621424 10.1002/joa3.12597 PMC8485794

